# Enhanced catalytic performance of palladium nanoparticles supported on novel N and F co-doped bio-carbon material for 5-hydroxymethylfurfural conversion

**DOI:** 10.1371/journal.pone.0320604

**Published:** 2025-03-25

**Authors:** Dandan Li, Feichao Miao, Jinhua Chen, Zhibing Liu, Zhiyuan Wang, Yang Wang

**Affiliations:** 1 School of Chemical and Blasting Engineering, Anhui University of Science and Technology, Huainan, China; 2 Anhui Key Laboratory of Explosive Energy Utilization and Control, Huaibei, China; Universiti Teknologi Petronas: Universiti Teknologi PETRONAS, MALAYSIA

## Abstract

A novel N, F-co-doped bio-carbon material derived from cellulose was successfully developed *via* a convenient and simple strategy. The obtained bio-carbon material was characterized with various techniques. It is found that the addition of N, F precursors can benefit the formation of porous structure in bio-carbon during thermal pyrolysis and N, F dopants have significant effect on electronic structure of native carbon atoms, which could regulate the adsorption property and catalytic activity of obtained bio-carbon material. Furthermore, Pd NPs supported on the N, F-co-doped bio-carbon showed excellent catalytic activity and selectivity toward the aerobic oxidation of biomass-derived 5-hydroxymethylfurfural to 2,5-furandicarboxylic acid in aqueous media under mild conditions. N, F dopants on bio-carbon material played an important role in the oxidation reaction.

## Introduction

It is well established that carbon materials are extensively employed in both academic research and industrial production [1]. For example, graphene [2], activated carbon (AC) [3] and carbon nanotube [4] are some of the typical carbon materials that promoted the social development in terms of energy and technology. However, those commercial carbon materials are mainly manufactured from non-renewable resources, such as petroleum and coal products. With the large depletion and environmental crises, there is a clear and urgent need to seek environmentally benign, low-cost and sustainable carbon sources. Biomass (e.g., lignin, cellulose) originating from the photosynthesis of carbon dioxide, water and solar energy is a rich and renewable source and an ideal raw material to prepare carbon materials [5,6]. Moreover, as a result of abundant surface functional groups, biomass-based carbon materials (bio-carbon) appear to possess more promising applications in comparison with conventional carbon materials [7,8], and has been applied in various fields, such as adsorption [9], energy storage [10] and catalysis [11]. For example, chitosan derived carbon material owned self-doped N/O structure would benefit the dispersion of metal nanoparticles compared to commercial carbon materials [12].

On the other hand, heteroatom doping has been deemed as an effective strategy improve the performance of carbon materials in various fields. Heteroatom doping could modulate carbon materials for the difference between dopants and C atoms in atomic type, atomic radius and electronegativity [13–15]. For example, introducing N species into carbon materials could enhance the hydrophilicity of carbon material,provide metal anchoring sites and affect the electronic properties of transition metals [16,17]. The strong electronegativity of F atom enhances charge transfer and electron redistribution between transition metals and carbon substrate [18].

Biomass can not only be utilized as carbon source of carbon materials but also transferred into valuable biofuels and industrial chemicals [19]. For example, 2,5-furandicarboxylic acid (FDCA), a potential alternative to terephthalic acid in the production of polyamides, polyesters, and polyurethanes, can be synthesized by oxidation of 5-hydroxymethylfurfural (HMF), a typical biomass- derived platform molecule [20–22]. Heterogeneous catalytic oxidation of HMF to FDCA with molecular oxygen (O_2_) in aqueous media is regarded as the most efficient and environmentally friendly method to produce FDCA, especially supported noble metal catalysts with high activity and selectivity. Many supported noble metal (Au [23], Pd [24], Ru [25], etc.) nanoparticles (NPs) have been reported and exhibited excellent performance in the conversion.

Recently, researches about carbon supported catalysts applied in the oxidation reactions have been reported [26–28]. Effects of different dopants on carbon materials were also investigated. Petra Jongh and co-workers reported the carbon-supported Au NPs in the oxidation of HMF [29]. It is found that the activity and selectivity of the catalyst strongly depend on the surface properties of carbon support. Positively charged carbon support has a better adsorption capability of hydroxyl ions, which act as a co-catalyst for Au NPs and thus enhance the activity of Au catalyst. In contrast, negatively charged carbon support repel hydroxyls ions and the intermediate monoacid anions, thus result in a high selectivity toward 5-hydroxymethylfurancarboxylic acid. In this regard, N and F doped carbon materials seems to be a promising catalyst support for the oxidation of HMF in aqueous phase. N-doped materials possess great dispersion in water, which is important for reactions in water phase [16,30,31]. And the strong electronegativity of F would induce neighbor carbon atoms with a strong positive charge [32–36]. However, to the best of our knowledge, there has been no report about F-doped carbon material as a catalyst support for aerobic oxidation.

Herein, we report a convenient and scalable method for fabricating Pd -based N,F-co-doped bio-carbon catalysts by simply pyrolyzing cellulose, polytetrafluoroethylene (PTFE) and melamine. Addition of PTFE and melamine could not only introducing N, F atoms into bio-carbon but also benefit the formation of porous structure during pyrolysis. The obtained catalyst exhibited high catalytic activities for the aerobic oxidation of HMF.

## Materials and methods

### Material

All chemicals were purchased from commercial suppliers. Micro-cellulose with an approximate length of 60 μm, palladium chloride (PdCl_2_), polytetrafluoroethylene (PTFE), melamine were purchased from Macklin Biochemical Technology Co., Ltd. (Shanghai, China) and used as received.. Sodium borohydride (NaBH_4_) were purchased from Sigma-Aldrich (St. Louis, MO, USA).

### Preparation of carbon materials

Cellulose (3 g), PTFE (700 mg), melamine (700 mg) were grounded in a mortar for 30 min before pyrolysed at 700°C under N_2_ atmosphere for 2 h with a heating rate of 5°C min^ − 1^. The obtained bio-carbon material was washed with deionized water (3 × 15 ml) and ethanol (3 × 15 ml). Finally, the bio-carbon were dried at 100°C overnight and denoted as NFBC.

Bio-carbon without dopants (BC) was prepared using only cellulose and pyrolyzed at 700 °C under the same conditions as NFBC.

### Pd catalysts preparation

Carbon materials (1 g), PdCl_2_ (10 mg) was added into 20 mL deionized water, after stirred for 5 h at room temperature, freshly prepared NaBH_4_ (10 mg) solution was added and the mixture was further stirred for another 1 h. The solid was collected by filtration and washed with water followed by ethanol and dried in vacuum at 60 °C for 5-6 h. This black powder was denoted as Pd/NFBC and Pd/BC, respectively.

### Characterization of catalysts

The surface morphology of the catalyst was characterized by transmission electron microscopy (TEM) with a PHILIPS Tecnai 12 microscope at 120kv. Energy Dispersive X-ray Spectroscopic analysis (EDS) was performed with a JEM-2010(HR) TEM at 200kV. X-ray photoelectron spectroscopy (XPS) was performed on a ESCALAB 250Xi spectrometer with a Al Kα X-ray source (1350 eV of photons). Inductively coupled plasma mass spectrometry (ICP-MS) was analyzed on Optima 7300 DV to detect Pd content. The samples were digested with HCl, HNO_3_ and HF solution at 180 °C for 30 min to operate ICP-MS analysis. XRD was taken with Panalytical X’Pert Pro with a Cu Kαradiation at 40 Kv in the scanning range from 10° to 80° and further analysed with ICDD (International Centre for Diffraction Data) as database. O_2_-TPD data were obtained on a Micromeritics AutoChem II S3 2920 instrument. The sample was first preheated at 200 °C for 1 h and then swept by 3% O_2_/He flow after cooled down. The O_2_-TPD signals were collected when the sample was heated in He stream to 800 °C with a rate of 10 °C/min.

### Oxidation of HMF

The catalytic oxidation of HMF was carried out in a Schlenk tube. Typically, HMF (0.05 mmol), catalysts, K_2_CO_3_ and deionized water (3 ml) were charged into a 15 ml Schlenk tube. The tube was sealed and purged with O_2_ four times to replace air. Then the tube was settled in an oil bath at a setting temperature and maintained at this temperature with continuous stirring for 12 h. After reaction, the mixture was neutralized with 1M HCl. The catalysts were recovered from the aqueous phase by centrifugation and washed with water (3 × 5 ml). The liquid part was diluted with MeOH and analysed by HPLC.

## Results and discussion

The catalysts Pd/NFBC and Pd/BC were synthesized using a simple and convenient process of physical mixing, carbonization and chemical reduction. Cellulose, an abundant biomass on earth, was chosen as carbon precursor. Melamine was attributed to N source while PTFE powder was attributed to F source.

### Catalysts characterization

A series of characterizations were carried out to explore the structure and surface chemical state of the catalysts, such as XRD, SEM, TEM, EDS, BET and XPS.

**Fig 1:**
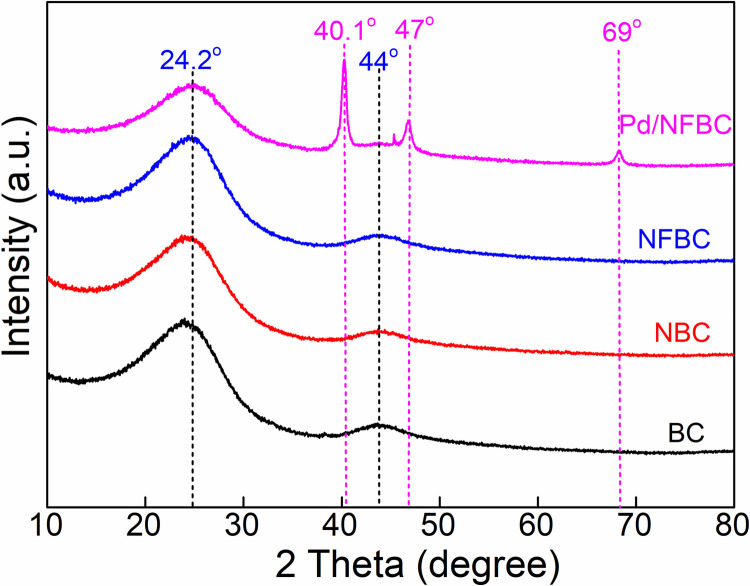
XRD patterns of BC, NBC, NFBC and Pd/NFBC.

First, powder X-ray diffraction (XRD) was performed (Fig 1). A broad characteristic peak at 2θ=24.2° and 2θ=43.4° corresponding to (002) diffraction of diffracted amorphous carbon (PDF#01–0604).. In comparison, no obvious difference was found between XRD spectra of NFBC, NBC and BC, indicating N, F dopants have little effect on the crystalline structure of carbon materials. As for Pd/NFBC, peaks at 40°, 47°, and 69° assignable to (111), (200), and (220) crystal planes of Pd lattice (PDF#01–1310) were observed.

**Fig 2:**
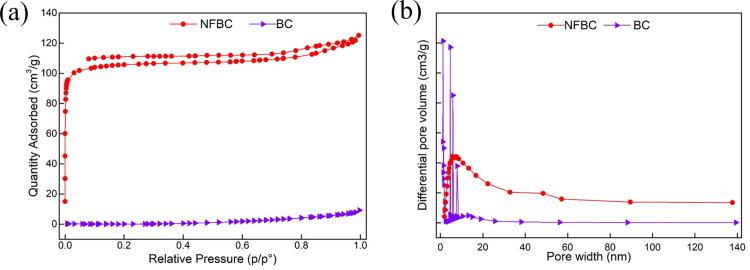
(a) N_2_ adsorption−desorption isotherms and (b) pore size distribution of BC and NFBC.

The surface morphology of NFBC and BC is imaged by scanning electron microscopy (SEM). As shown in S1 Fig and S2 Fig in S1 File, NFBC and BC both showed a columned structure with heavy crumpling features. However, no obvious pore structure was observed in both carbon materials. To make sure if the obtained carbon materials were porous, Brunauer−Emmett−Teller (BET) was carried out. The results were displayed in Table 1 and Fig 2. To our great surprise, though both NFBC and BC are not materials that possess a large amount of pores, NFBC has a much larger surface area than BC, which would be attributed to the N,F-doping (Table 1). Furthermore, The N_2_ adsorption/desorption isotherms of NFBC indicate that NFBC possesses the typical type I isotherm with a sharp increase at the low relative pressure, indicating the existence of a large number of micro-pores in NFBC (Fig 2). Meanwhile, curve of BC indicates BC possesses the typical Ⅱ isotherm with low absorbed gas volume, indicating BC is nonporous material. The BET results demonstrated that addition of melamine and PTFE can facilitate template-free formation of porous structures in bio-carbon material, which might result from the gas evolution during thermal decomposition of melamine and PTFE.

**Table 1:** Specific surface areas.

Sample	Specific surface area(m^2^/g)	Pore volume (cm^3^/g)
NFBC	424.14	0.19
BC	0.44	0.02

**Fig 3:**
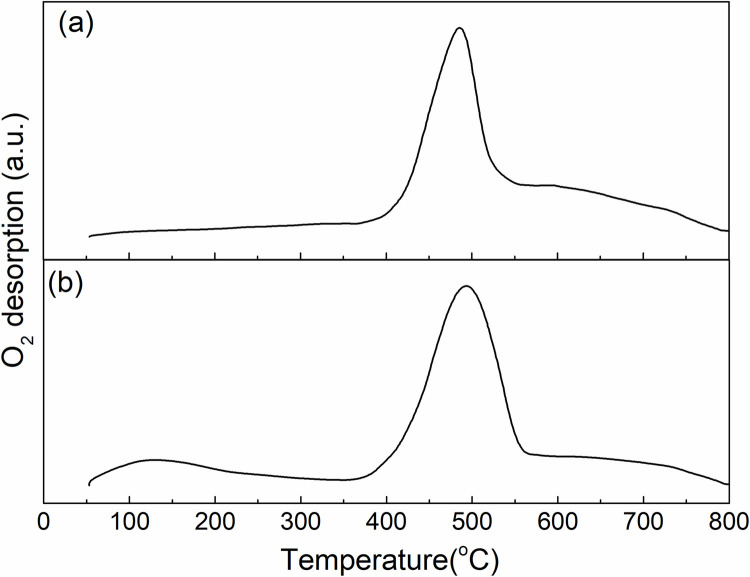
O_2_-TPD profiles of (a) Pd/BC and (b) Pd/NFBC.

Temperature-programmed desorption of O_2_ (O_2_-TPD) over Pd/NFBC and Pd/BC were also studied. As presented in Fig 3, two main types of oxygen species could be identified, according to the oxygen desorption temperatures in the O_2_-TPD profiles. The desorption peak at higher temperature (350–515^o^C) can be ascribed to the lattice oxygen species bonded to Pd^2 +^ cations, indication Pd NPs in both Pd/NFBC and Pd/BC were oxidized during the pretreatment process at 500 °C. The desorption peak at lower temperature (≤350^o^C) can be assigned to the surface active oxygen species and molecular oxygen adsorbed on the surface. NFBC obvious show higher concentration of surface active oxygen species while nearly no surface active oxygen species were detected over BC. The results clearly indicated that Pd/NFBC has a better O_2_ adsorption capacity and higher O_2_ activation ability.

**Fig 4:**
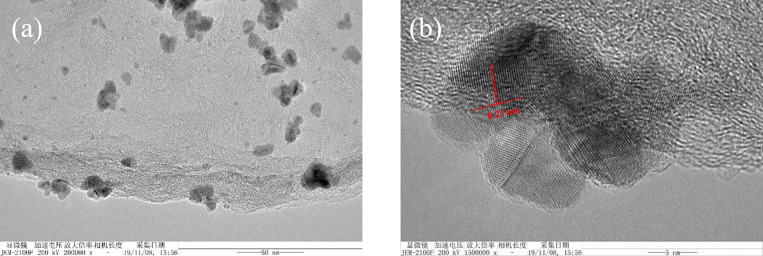
(a) TEM and (b) HRTEM images of Pd/NFBC.

**Fig 5:**
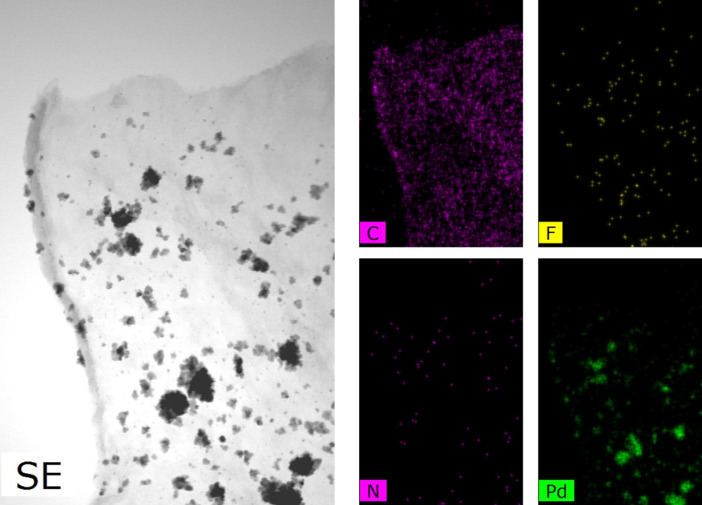
Element mapping of Pd/NFBC.

Transmission electron microscopy (TEM) images of Pd/NFBC show that most Pd NPs were uniformly distribute on the carbon surface (Fig 4a), although some aggregation was also observed. The aggregation might because of robust reduction of NaBH_4_. The uniform distribution of Pd NPs provides more contact opportunity between the active sites and the reactants. Further high-resolution (HR)TEM analysis revealed a highly integrated nanostructure of Pd NPs (Fig 4b). The well-resolved lattice spacing of 0.23 nm corresponding to the (111) of the metallic Pd can be perceived. EDX mapping images of the catalyst confirmed the successful doping and evenly distribution of F and N atoms on NFBC surface (Fig 5).

**Fig 6:**
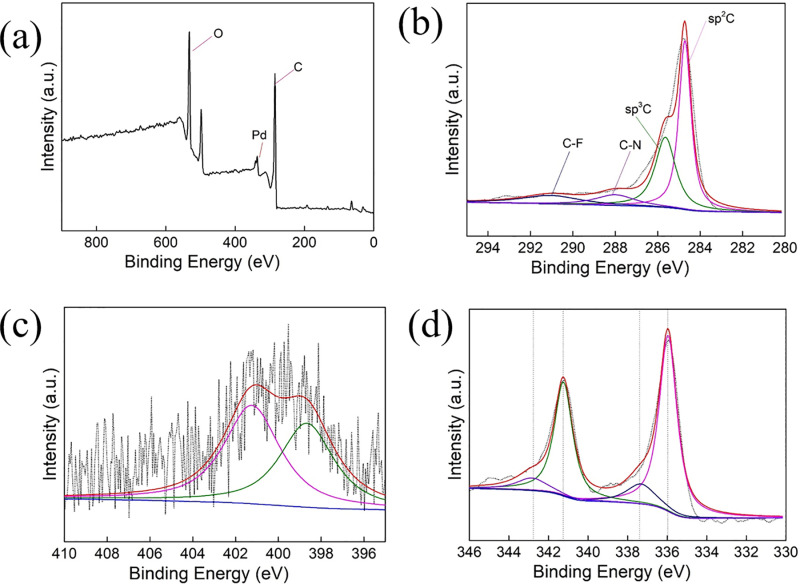
a) XPS full spectra of Pd/NFBC; b) XPS C 1s spectra of Pd/NFBC; c) XPS N 1s spectra of Pd/NFBC, and d) XPS Pd 3d spectra of Pd/NFBC.

XPS spectra were recorded to investigate the chemical state of surface atoms in Pd/NFBC. As seen in the wide scan spectra (Fig 6a), Pd/NFBC catalysts contain C, N, and Pd, which is also confirmed using the EDX spectrum (Fig 5). However, the peak of F is not obvious because of its low content. There is a sharp decrease of N, F content, compared with the ratio between cellulose, PTFE and melamine before pyrolysis. The results suggest that N and F may be released as a result of thermal gas evolution. And the thermal gas serves as a soft template of porous structure, which leads to the formation of micro-pores in bio-carbon.

The C 1s XPS spectra consist of four peaks located at 284.6 eV, 285.9 eV, 288.0 eV and 291.6 eV corresponding to sp^2^C, sp^3^C, C–N and C–F species, respectively (Fig 6b). As shown in Fig 6b, both N and F dopants induce neighboring C atoms positive charge, which was confirmed to be important reason for enhancing the activity and selectivity of carbon-base catalysts in oxidation of HMF [29]. N 1s spectrum of Pd/NFBC is shown in Fig 6c. Only two components can be deconvoluted from the spectrum, representing pyridinic N (398 eV) and graphitic N (401 eV). According to the Pd 3d region spectrum, Pd^0^ and Pd^2 +^ coexist on the surface of Pd/NFBC (Fig 6d) and the calculation from Pd 3d peaks showed that Pd element mainly exists in Pd^0^ state (S1 Table in S1 File). A small amount of Pd^2 +^ species might due to oxidation of Pd^0^ under ambient conditions. The peaks at 335.8 eV and 339.9 eV were assigned to the Pd 3d_5/2_ and Pd 3d_3/2_ transitions, respectively. Pd/NBC and Pd/FBC were also characterized by XPS (S3 Fig in S1 File). No C–F species was found in Pd/NBC and no C–N specie was found in Pd/FBC. Pd element mainly in Pd^0^ state (S1 Table in S1 File). However, the proportion of Pd specie in Pd/FBC is obviously lower than that in Pd/NBC, indicating F dopants might benefit formation of metallic Pd.

### Catalysis test

The catalytic activity of Pd/NFBC and Pd/BC was examined in the aerobic oxidation of HMF to FDCA under atmospheric O_2_ with water as a green solvent. In this reaction, K_2_CO_3_ is used as base source. Pd/NFBC exhibited higher activity towards the oxidation of HMF (Table 2, Entries 1-2), i.e., HMF conversion of 96% and FDCA selectivity of 93%. It is obvious that N, F dopants enhanced the activity of bio-carbon supported Pd catalysts. Combined with the characterization of the catalysts and previous researches [9], introduction of N, F dopants will lead to charge redistribution, C in NFBC has much larger compensating positive charge compared with C in carbon material without dopants [37], as shown in the C 1s XPS spectra. The unique electronic properties of carbon modified by the N and F dopant make it a superior position for adsorption of reactive oxygen species such as peroxide and hydroxyl ions, thus accelerate the oxidation process [29].

**Table 2:** Study of HMF Oxidation^a^.

Entry	Cat.(%)	K_2_CO_3_(equiv.)	T(°C)	Con.(%)^b^	Sel.(%)^b^
1	Pd/NFBC(1.0)	2.0	100	96	93
2	Pd/BC(1.0)	2.0	100	60	67
3	Pd/NFBC(1.0)	2.0	80	81	79
4	Pd/NFBC(1.0)	2.0	120	95	92
5	Pd/NFBC(1.0)	4.0	100	95	66
6	Pd/NFBC(1.0)	3.0	100	97	85
7	Pd/NFBC(1.0)	1.0	100	56	79
8	Pd/NFBC(0.7)	2.0	100	88	87
9	Pd/NFBC(0.5)	2.0	100	52	84
10^c^	Pd/NFBC(1.0)	2.0	100	63	46

^a^Reaction conditions: HMF (0.2 mmol), Pd catalyst, K_2_CO_3_, H_2_O (3 mL), O_2_ balloon, 12h. Catalyst amount based on Pd.

^b^Detected by HPLC.

^c^Air balloon.

Besides, the porous structure played an important role in the oxidation process. The increase of pore structure in carbon materials would lead to the increase of O_2_ adsorbed on the catalyst. More contact opportunity between O_2_ and the active sites would further accelerate the oxidation process. As shown in the BET results, NFBC has a much larger surface area and pore volume than BC. That would be another reason of the high activity of Pd/ NFBC. And the O_2_-TPD results also confirmed that more O_2_ was adsorbed and activate by Pd/NFBC.

The reaction conditions of HMF oxidation was further optimized. First, reaction temperature played an important role in the oxidation process. Both conversion of HMF and selectivity to FDCA decreased at a lower temperature, while a higher temperature did not have significant effect on the result (Table 2, Entries 3-4 and S4 Fig in S1 File). Base is necessary in the oxidation of HMF catalysed by Pd-based catalysts. In Pd catalysed oxidation of HMF, the combination of base and HMF to form an intermediate is the first step. Further, the intermediate is adsorbed on the catalyst for further dehydration and oxidation, and finally the target product FDCA is obtained [38]. K_2_CO_3_ was chosen as a weak base in this study for its abundance and easy-handling proprieties. Strong base such as NaOH might increase pH of the reaction mixture thus promoting degradation of HMF and lead to low yield of FDCA. Then different base amounts were also examined. No FDCA was detected without base. Only 56% HMF was converted with 1.0 equiv. K_2_CO_3_ and the selectivity to FDCA was 79%. Increasing base amount led to higher conversion of HMF (Table 2, Entries 5-7 and S4 Fig in S1 File). It should be noted that excess K_2_CO_3_ also had negative effect on this reaction. This trend was much more apparent with further increase of K_2_CO_3_ amount. With the K_2_CO_3_ amount was increased from 2.0 equiv. to 3.0 equiv., selectivity to FDCA was unexpectedly decreased. Increased K_2_CO_3_ amount would lead to increase of alkaline content, thus mono-acid intermediate remained in salt form, which prevents adsorption of the carboxylic acids on the catalyst and further oxidation of the intermediate. 2.0 equiv. K_2_CO_3_ was considered optimum for HMF oxidation reaction.

At last, different catalyst amounts were examined (Table 2, Entries 8-9 and S4 Fig in S1 File). 0.7% catalyst gave 88% conversion of HMF, 75% selectivity of FDCA and 0.5% catalyst gave 52% conversion and 69% selectivity. In general, the higher the catalyst amount, the higher HMF conversion and selectivity of FDCA. This could be explained by the increased of catalytically active sites number and increased contact opportunities between the reactants and the active sites. Therefore, the optimized conditions for oxidation of HMF to FDCA was 1.0% catalyst with 2.0 equiv. K_2_CO_3_ at 100°C.

The high catalytic activity of Pd/NFBC inspired us replace O_2_ with air in the aerobic oxidation of HMF. To our delight, Pd/NFBC also performed well in air condition (Table 2, Entry 10). HMF conversion of 63% and FDCA selectivity of 46% were observed. The lower conversion and selectivity were because of the lower O_2_ concentration in air.

### Catalyst reusability

**Fig 7:**
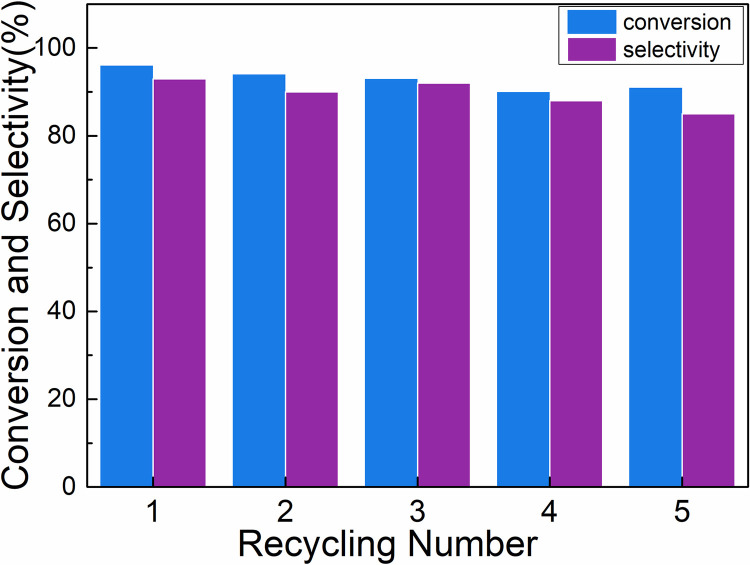
Recycle usage of the Pd/NFBC for oxidation of HMF.

Finally, reusability of Pd/NFBC was studied. After reaction, Pd/NFBC can be facilely recovered by centrifugation and reused in next run. Catalysts were washed with deionized water for 3 times and directly used in next cycle without further drying. As shown in Fig 7, Pd/NFBC exhibited excellent reusability and can be reused for at least 4 cycles without obvious decrease in conversion and selectivity. After 5 runs, the conversion and the selectivity both decreased. Slight leaching of Pd was detected by ICP characterization (S2 Table in S1 File) and agglomeration of Pd NPs was observed with TEM. The leaching and agglomeration of Pd may be the main reasons for the drop of catalytic performance.

## Conclusions

In summary, a novel biomass-derived carbon material with N, F dopants was synthesized by carbonization of cellulose, PTFE and melamine. Pd NPs supported on the obtained bio-carbon material showed excellent activity and selectivity in the aerobic oxidation of HMF to FDCA. The as-prepared Pd/NFBC catalyst showed better catalytic activity than Pd/BC. It is found that N, F dopants can not only benefit the formation of porous structure during carbonization process but also affect the chemical state of carbon surface. N and F dopants induce neighboring C atoms positive charge and better O_2_ adsorption capacity, thus promote the catalyst performance in the aerobic oxidation of HMF. Therefore, the low-cost and environmentally friendly dual doped carbon material hold great promise for a wide range of catalytic transformation.

## Supporting information

S1 File Supporting information.
(DOCX)
