# Supplementary material for: Enhanced catalytic performance of palladium nanoparticles supported on novel N and F co-doped bio-carbon material for 5-hydroxymethylfurfural conversion
Source: PLoS One. 2025 Mar 25;20(3):e0320604. doi: 10.1371/journal.pone.0320604 (PMC11936169; doi:10.1371/journal.pone.0320604)
Supplement: S1 File — (DOCX) [file pone.0320604.s001.docx]

**Supporting Information**

**Enhanced catalytic performance of palladium nanoparticles supported on novel N and F co-doped bio-carbon material for 5-hydroxymethylfurfural conversion**

**Dandan Li^1,2^,** **Feichao Miao ^* 1,2^, Jinhua Chen ^2^, Zhibing Liu ^2^, Zhiyuan Wang^1^ and Yang Wang^1^**

^1^ School of Chemical and Blasting Engineering, Anhui University of Science and Technology, Huainan, China,232001,

^2^ Anhui Key Laboratory of Explosive Energy Utilization and Control, Huaibei, China,235000

* Feichao Miao

E-mail: [miaofeichao@qq.com](mailto:miaofeichao@qq.com) (FM)

# SEM images of the carbon materials


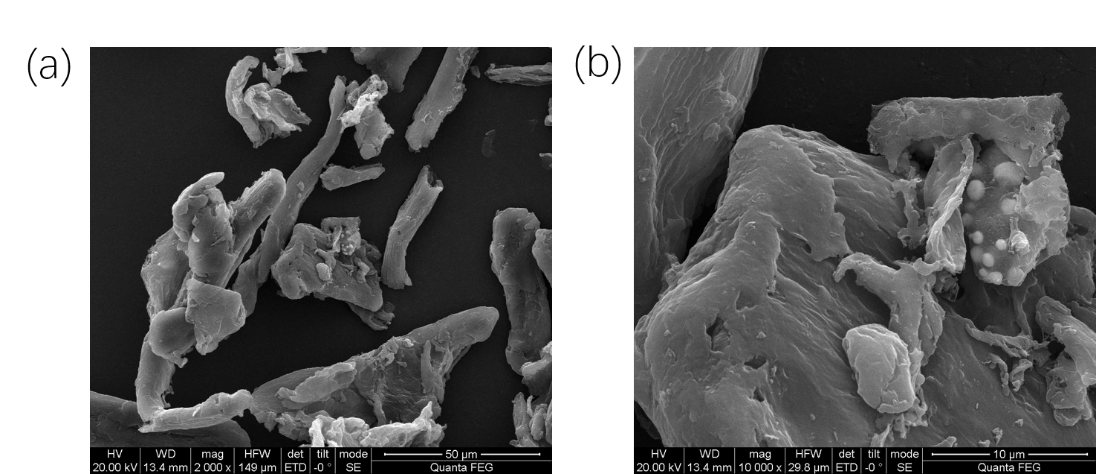


S1 Fig. SEM images of NFBC


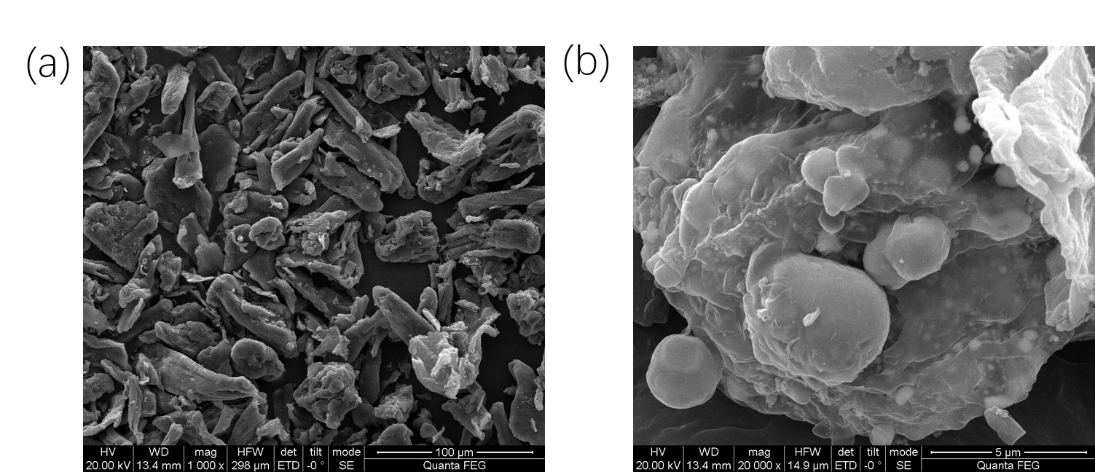


S2 Fig. SEM images of BC

# XPS spectra of Pd/NBC and Pd/FBC


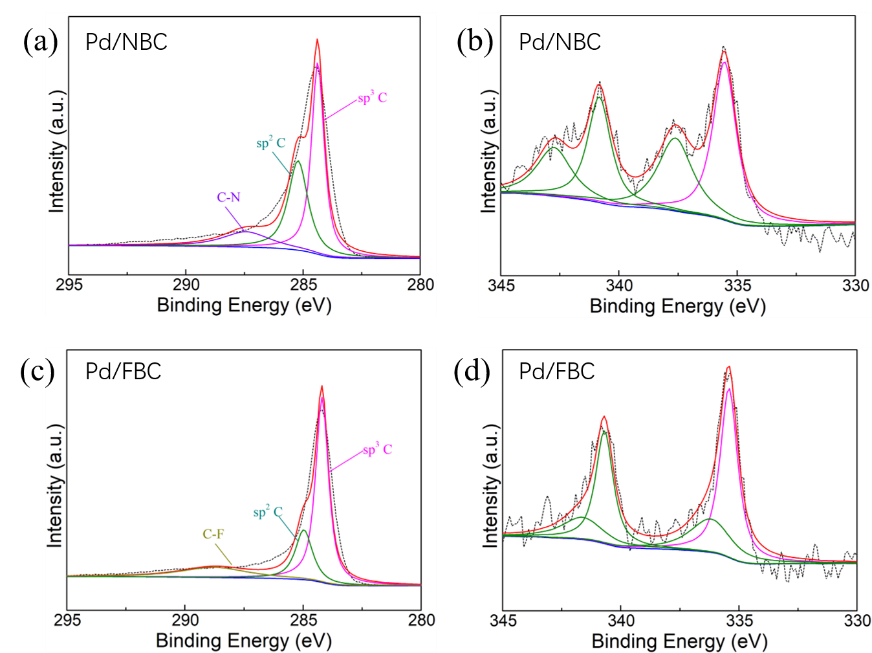


S3 Fig. XPS spectra of Pd/NBC and Pd/FBC

# Results of XPS Characterization of Pd/NFBC

S1 Table. Results of XPS Characterization for Pd/NFBC

| catalyst | distribution of Pd species | | Pd^0^3d_5/2_ (eV) |
| --- | --- | --- | --- |
|  | Pd^0^ | Pd^2+^ |  |
| Pd/NFBC | 83 | 17 | 335.8 |
| Pd/NBC | 59 | 41 | 335.53 |
| Pd/FBC | 79 | 21 | 335.42 |

# ICP of Pd/NFBC and recycled Pd/NFBC

S2 Table ICP of Pd/C_6_F_13_-Cell and recycled Pd/C_6_F_13_-Cell

| Cat. | ICP data (mg/g_Cat._) |
| --- | --- |
| Pd/NFBC | 1.0032 |
| Pd/NFBC reused 5 runs | 0.8977 |


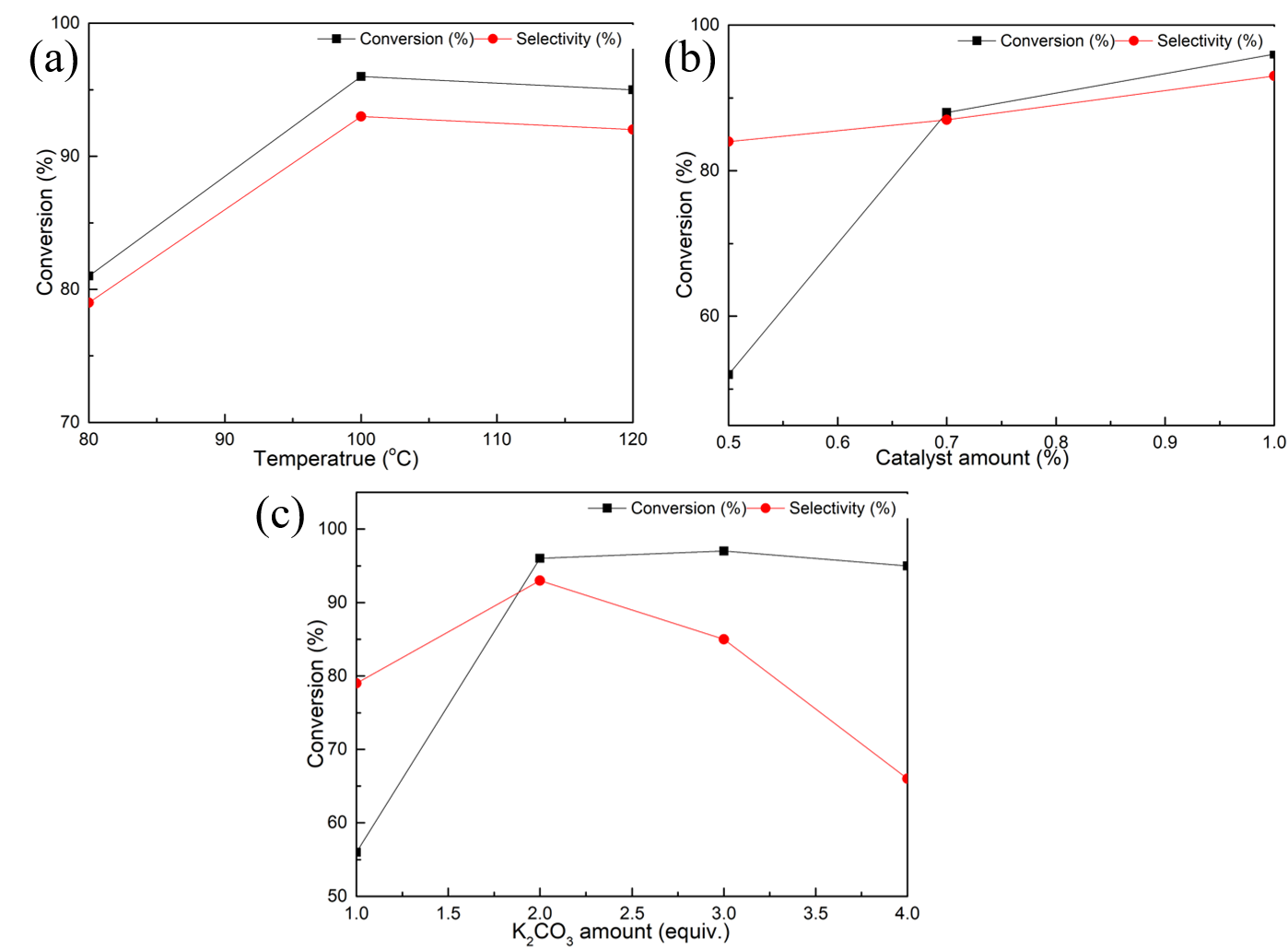


S4 Fig. plots of HMF oxidation conversion and selectivity versus different factors.

# TEM images of reused Pd/NFBC


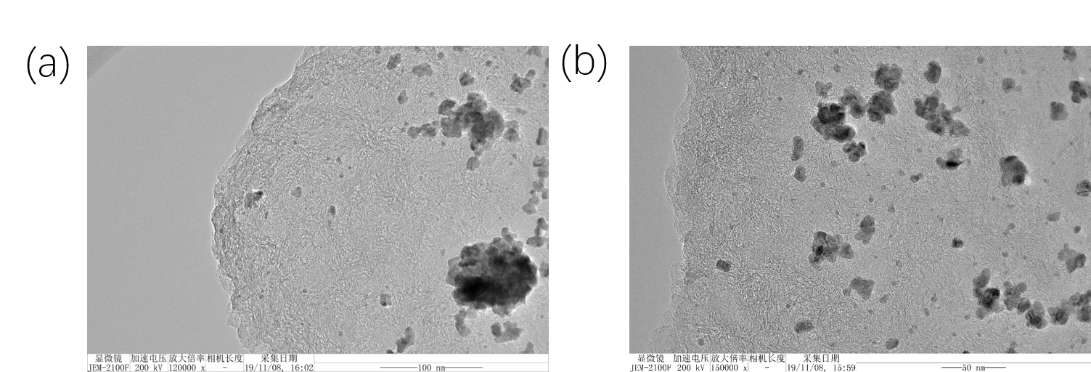


S5 Fig. TEM images of reused Pd/NFBC
